# Relationship between e-cigarette point of sale recall and e-cigarette use in secondary school children: a cross-sectional study

**DOI:** 10.1186/s12889-016-2968-2

**Published:** 2016-04-14

**Authors:** Catherine Best, Farhana Haseen, Winfried van der Sluijs, Gozde Ozakinci, Dorothy Currie, Douglas Eadie, Martine Stead, Anne Marie MacKintosh, Jamie Pearce, Catherine Tisch, Andy MacGregor, Amanda Amos, John Frank, Sally Haw

**Affiliations:** School of Health Sciences, University of Stirling, Stirling, FK9 4LA UK; Child and Adolescent Health Research Unit, School of Medicine, University of St Andrews, St Andrews, KY16 9TF UK; School of Medicine, University of St Andrews, St Andrews, KY16 9TF UK; Institute for Social Marketing, School of Health Sciences, University of Stirling, Stirling, FK9 4LA UK; Centre for Research on Environment Society and Health, School of GeoSciences, University of Edinburgh, Edinburgh, EH8 9XP UK; ScotCen Social Research, Edinburgh, EH2 4AW UK; The Usher Institute of Population Health Sciences and Informatics, College of Medicine and Veterinary Medicine, University of Edinburgh, Edinburgh, EH8 9AG UK

**Keywords:** E-cigarettes, Vaping, Adolescents, Advertising, Point of sale display, Smoking, Tobacco control

## Abstract

**Background:**

There has been a rapid increase in the retail availability of e-cigarettes in the UK and elsewhere. It is known that exposure to cigarette point-of-sale (POS) displays influences smoking behaviour and intentions in young people. However, there is as yet no evidence regarding the relationship between e-cigarette POS display exposure and e-cigarette use in young people.

**Methods:**

This cross sectional survey was conducted in four high schools in Scotland. A response rate of 87 % and a total sample of 3808 was achieved. Analysis was by logistic regression on e-cigarette outcomes with standard errors adjusted for clustering within schools. The logistic regression models were adjusted for recall of other e-cigarette adverts, smoking status, and demographic variables. Multiple chained imputation was employed to assess the consistency of the findings across different methods of handling missing data.

**Results:**

Adolescents who recalled seeing e-cigarettes in small shops were more likely to have tried an e-cigarette (OR 1.92 99 % CI 1.61 to 2.29). Adolescents who recalled seeing e-cigarettes for sale in small shops (OR 1.80 99 % CI 1.08 to 2.99) or supermarkets (OR 1.70 99 % CI 1.22 to 2.36) were more likely to intend to try them in the next 6 months.

**Conclusions:**

This study has found a cross-sectional association between self-reported recall of e-cigarette POS displays and use of, and intention to use, e-cigarettes. The magnitude of this association is comparable to that between tobacco point of sale recall and intention to use traditional cigarettes in the same sample. Further longitudinal data is required to confirm a causal relationship between e-cigarette point of sale exposure and their use and future use by young people.

## Background

There is increasing concern about the rising prevalence of e-cigarette use among children and young people in particular, that e-cigarette use is increasing among young people who have never smoked (never smokers) [1]. Although e-cigarettes are substantially less harmful than smoking, the long term health impact of e-cigarette use by never smokers and the long term consequences of dual use (e-cigarette and tobacco smoking) are uncertain [2]. In this context, it is important that we understand what factors predispose adolescents to initiate e-cigarette use.

It has been clearly demonstrated that increased availability of cigarettes is associated with higher consumption. For example, a higher tobacco retail outlet density in a young person’s home environment increases the probability of current smoking and ever smoking [3] and the same is true for current smoking in adults [4]. The effect of increased taxes on tobacco similarly reduces availability to young people and decreases consumption [5, 6]. Young people’s perceived ease of access to cigarettes has been shown to interact with the social desirability of smoking to reduce tobacco experimentation [7]. Point of sale (POS) displays of tobacco products influence young people to take up smoking. Cross sectional studies in the UK and New Zealand have shown that self-reported recall of tobacco POS is related to smoking susceptibility in young never smokers [8, 9] and that young people who notice tobacco POS are more likely to be current smokers [10]. A longitudinal study in the US found that young never smokers who recall tobacco POS are more likely to be smokers at 30 month follow up [11]. Studies of the effect of tobacco point of sale bans in various parts of the world have shown the reversibility of these effects. After the POS ban in Australia young people had reduced odds of being current smokers [12] and in Ireland young people’s perception of smoking prevalence dropped following the POS ban [13]. Systematic reviews have concluded there is a relationship between tobacco POS exposure and smoking related outcomes in young people [14–16]. It is plausible that POS displays of e-cigarettes may have similar influence.

The e-cigarette market is undergoing a period of substantial growth internationally [17] and the number of convenience-type stores stocking and displaying e-cigarettes is increasing rapidly [18, 19]. In Scotland, around 77 % of tobacco retailers sold e-cigarettes in 2014 and around 49 % had e-cigarette POS displays [20]. It is not known what impact the increase in retail availability and in-store promotion of e-cigarettes has on the likelihood of young people trying e-cigarettes. This study is the first to examine e-cigarette POS exposure and use in young people. It also compares the relationship between e-cigarette POS exposure and use with the well-characterised association between cigarette POS exposure and tobacco smoking. We cannot assume these relationships will be similar because the cigarette market and brands are far more established and more instantly recognisable to potential purchasers than the relatively recent and variable presence of e-cigarettes in the retail environment.

The regulatory environment for e-cigarettes is very different to that for tobacco. While most tobacco advertising and promotion is prohibited in the UK [21] and sale to under 18 year olds illegal, currently e-cigarettes are not subject to such regulation. This will change when the European Union’s Tobacco Products Directive (2014/40/EU), replacing Directive (2001/37/EC), comes into force in May 2016. After that time existing rules regarding the cross border advertising and promotion of tobacco products will apply to e-cigarettes. Therefore, until after May 2016 there are no specific legal constraints on how e-cigarettes are marketed across the EU. Even after this time it will be left to the discretion of member states whether they decide to impose age limits for purchase, restrictions on flavourings and/or POS promotion of e-cigarettes. For example, in Scotland  the Health (Tobacco, Nicotine etc. and Care) (Scotland) Bill was passed in March 2016. This prohibits e-cigarette sales to under 18s but does not ban outright POS displays. This creates a window of opportunity for the marketing and promotion of e-cigarettes.

Young people are not only exposed to e-cigarette adverts through POS displays but also from TV, billboards, print media and the internet. Experimental research has shown that exposure to e-cigarette TV adverts increases the likelihood that adolescents will intend to try e-cigarettes in the future [22].

This Scottish study examines the relationship between the recall of e-cigarette POS displays and children’s use and future intention to use e-cigarettes controlling for exposure to online advertising of e-cigarettes, e-cigarette advertising via other media, smoking status and demographic factors. In order to compare the size of effects we also examine the relationship of cigarette point of sale exposure to intention to smoke cigarettes in the same sample. To our knowledge information on young people’s recall of e-cigarettes in retail outlets and use and intention to use e-cigarettes has not been collected in any other survey to date so this is the first and currently the sole source of information on this important and policy relevant topic.

## Method

### Study

The data presented here were collected as part of an ongoing 6-year multi-modal study designed to assess the impact of Scottish legislation banning tobacco POS displays on young people’s smoking-related attitudes and behaviours [23]. The information on e-cigarette advertising exposure, e-cigarette use and smoking status were gathered through a school-based survey conducted in four purposively selected communities. Schools were selected to reflect two levels of urbanisation (urban vs. small town) and two levels of social deprivation (high vs. medium/low).

The results reported here are based on data collected between January and March 2015 after the implementation of the tobacco point of sale ban in large supermarkets but before its implementation in small shops. Questionnaires were administered to pupils in Secondary 1 through to Secondary 6 (age range 10.83–18.67 years, mean 14.71 sd 1.65) by teachers during class time under examination conditions. Information regarding the survey was sent out to parents two weeks prior to the survey date. Parents could withdraw their child from the survey by returning the opt-out form to the school. Young people could decline to take part in the survey or withdraw from the survey at any point. Ethical approval for the study, including the consent procedures, was obtained from the University of St Andrews, School of Medicine Ethics Committee.

### Analysis approach

This study aimed to examine the relationship between e-cigarette point of sale recall (in large supermarkets and small shops) and e-cigarette use. Previous e-cigarette use and intention to use e-cigarettes in the next 6 months were the dependent variables. E-cigarette POS recall in large supermarkets and small shops were the explanatory variables. Recall of other sources of e-cigarette advertisement was included in the adjusted models to control for the potentially confounding effects of exposure to other types of e-cigarette advertisement. In addition, demographic factors such as age, sex, and family affluence were included as control variables. Tobacco smoking is known to be a predisposing factor to e-cigarette use therefore previous tobacco use was also included in the adjusted models as a control variable in order to test whether e-cigarette POS sale influenced e-cigarette uptake in never smokers (never tried tobacco cigarettes).

#### Demographic variables

Respondents were asked their gender, ethnic group, and date of birth. Ethnic group was dichotomised to white ethnic group versus other responses. Individual family material well-being was assessed through the Family Affluence Scale (FAS) [24] which is a validated measure consisting of six questions (own bedroom, number of family cars, number of computers, number of family holidays abroad per year, owning a dishwasher and number of bathrooms). The FAS raw scores were transformed though categorical principal component analysis into single dimensional scores that were then divided into tertiles of high, medium, and low FAS scores. This asset based measure of family affluence has been shown to be strongly related to household income (eta squared around 0.30 in most countries [25]).

#### E-cigarette use

Respondents were asked whether they had heard of e-cigarettes and could respond ‘yes’, ‘no’, or ‘don’t know’. Respondents who answered ‘yes’ were directed to the question ‘which ONE of the following is closest to describing your experience of e-cigarettes?’, to which they could respond ‘I have never used them’, ‘I have tried them once or twice’, ‘I use them sometimes (more than once a month)’, or ‘I use them often (more than once a week)’. This variable was dichotomised to ‘ever tried-1’ versus ‘never tried-0’. The next question was ‘Do you think that you will try e-cigarettes in the next 6 months?’ to which they could reply ‘yes I do’, ‘no I don’t’ or ‘don’t know’. This response was also dichotomised to ‘yes’ -1 versus ‘no’ or ‘don’t know’-0. In the original coding of the e-cigarette variables participants who responded that they did not know what an e-cigarette was or missed this question were coded as ‘missing’ on all other e-cigarette questions. Another alternative coding method was employed to minimise missing data where participants who did not know what an e-cigarette was, were assumed not to have tried one and not to intend to try one. Results are presented for both versions of the e-cigarette dependent variables.

#### Cigarette smoking

Ever smoking was assessed with the question: ‘Have you ever smoked cigarettes, even if it is just one puff?’ to which they could respond ‘yes’ or ‘no’. Negative responses were used as our variable for ‘never smoked’. To assess intention to smoke respondents were asked ‘Do you think you will smoke a cigarette or hand-rolled cigarettes (roll-ups) at any time during the next year?’ To which they could answer ‘definitely yes’ ‘probably yes’ ‘probably not’ ‘definitely not’. This was dichotomised to ‘yes’-1 or ‘no’-0.

#### Advertising

Respondents were asked ‘During the past 30 days, have you noticed any adverts (e.g., shops, shopping centres, TV, radio, billboards, newspapers, magazines, etc.) for (a) cigarettes or hand rolled cigarettes (roll-ups), or (b) electronic cigarettes (e-cigarettes)?’ To which respondents could answer ‘Yes’, ‘no’ or ‘don’t know’. This response was also dichotomised to ‘yes’-1 versus ‘no’ or ‘don’t know’-0.

#### Internet advertising

Respondents were asked ‘When you are using the internet, how often do you see adverts for (a) tobacco products or pictures of people smoking, or (b) electronic cigarettes (e-cigarettes) or people smoking them?’ To this pupils could respond ‘I don’t use the internet’, ‘most of the time’, ‘some of the time’, ‘hardly ever’, ‘never’. Responses of ‘I don’t use the internet’ were recoded as ‘never’. All responses were dichotomised to ‘never’ -0 versus other responses-1.

#### Recall point of sale displays in supermarkets and shops

Respondents were asked whether in the past 30 days, when they had been in (a) large supermarkets and (b) small shops, they could remember seeing (i) cigarette or tobacco packs or (ii) electronic cigarettes displayed for sale. To this they could respond ‘yes’, ‘no’ or ‘don’t know’. This response was also dichotomised to ‘yes’ -1 versus ‘no’ or ‘don’t know’-0.

### Analysis

Data analysis and imputation was conducted in Stata version 14. Analysis was by logistic regression with standard errors adjusted for clustering by school. Where the prevalence of the outcome was low (i.e., near to 10 % for dependent variable ‘intention to try e-cigarettes' -Table 3 and dependent variable intention to try cigarettes-Table 4), the alpha value was adjusted to 0.01 [26] and therefore the 99 % confidence interval for the odds ratio is presented.

Interactions between demographic variables and other predictors were explored for all models and interaction terms retained in the models only if the interaction term was significant.

To test how robust the results were across different methods of handling missing data, we used multiple imputation by chained equations to fully impute the dataset. Multiple imputation by chained equations is an iterative process that imputes multiple variables using a series of univariate chained equations with fully conditional specification of prediction equations. It is based on Monte Carlo simulation techniques for sampling from complicated multivariate distributions. Auxiliary variables were included in the imputation model: whether parents were employed, receipt of free school meals and area level deprivation. A uniform prior distribution was assumed. A burn in of 10 iterations was employed and this was confirmed to lead to convergence through examination of trace plots. Twenty imputations were initially performed and adding additional imputations up to a total of 100 did not significantly change the parameter estimates.

## Results

The response rate to the survey was 87 % and a total sample of 3808 pupils was achieved. Table 1 shows the frequencies for the demographic and outcome variables. In this sample, 19.8 % of respondents reported having ever smoked. This included 4 % (148/3725) who were current smokers. Amongst young people who had ever tried smoking, 60.0 % (437/728) indicated that they had also tried an e-cigarette, while 29.9 % (215/718) said they intended trying one in the next 6 months. Amongst never smokers, only 8.6 % (252/2942) had ever tried an e-cigarette, while 2.7% (78/2896) intended to try one in the next 6 months.

**Table 1:** Participants’ characteristics

	n (valid %)	Missing data n (%)
Gender - female	1884 (49.7 %)	16 (0.4 %)
Ethnic group - white	3480 (92.3 %)	39 (1.0 %)
Family affluence scale - low	1268 (34.2 %)	105 (2.8 %)
Family affluence scale - medium	1157 (31.2 %)	105 (2.8 %)
Family affluence scale - high	1278 (34.5 %)	105 (2.8 %)
Never smoked tobacco	3001 (80.2 %)	64 (1.7 %)
Tried an e-cigarette	699 (22.7 %)	729 (19.1 %)
Tried an e-cig recoded	699 (18.8 %)	84 (2.2 %)
Intends to try e-cigarettes	296 (9.8 %)	785 (20.6 %)
Intends to try e-cig recoded	296 (8.1 %)	140 (3.68 %)
Recall e-cig supermarket - yes	1086 (29.2 %)	82 (2.2 %)
Recall e-cig small shop - yes	1014 (27.7 %)	83 (2.2 %)
Recall internet e-cig ads - yes	2550 (68.5 %)	86 (2.3 %)
Recall other e-cig ads - yes	1694 (45.5 %)	85 (2.2 %)
Intention to smoke in next year- definitely/probably yes	448 (12.1 %)	97 (2.5 %)
Recall cig supermarket - yes	2918 (78.1 %)	70 (1.8 %)
Recall cig small shop - yes	2736 (73.4 %)	79 (2.1 %)
Recall internet cig ads - yes	2459 (66.1 %)	84 (2.2 %)

### Recall of e-cigarette POS displays and previous use of e-cigarettes

In the unadjusted model, in which the predictor variables were individually regressed on e-cigarette experience, all types of e-cigarette advert recall were significantly associated with having tried an e-cigarette (Table 2 unadjusted model). In other words, recalling any type of e-cigarette advert or display was associated with an increase in odds of having tried an e-cigarette. In the adjusted model 1 (Table 2) when smoking experience, all types of e-cigarette advert recall and demographic variables were included in the model, only recall of e-cigarette POS displays in small shops and online remained significant predictors of previous e-cigarette use. That is, recall of e-cigarette POS in small shops was independently associated with e-cigarette ever use when recall of other types of e-cigarette advertising was controlled. Recall of other types of e-cigarette advert (e.g., in print media, TV or billboards) was not associated with previous e-cigarette use in the adjusted model. As anticipated, having never smoked tobacco was a significant protective factor against having tried e-cigarettes. Young people who had never smoked tobacco were much less likely to have tried e-cigarettes than those who had tried tobacco. In addition, older respondents were more likely to have tried e-cigarettes than younger ones.

**Table 2:** Logistic regression on e-cigarette ever use- adjusted for clustering within school

	Unadjusted model	Adjusted model 1	Adjusted model 2 recoded e-cig variable	Adjusted model 3 fully imputed m = 20
	OR (99 % CI)	OR (99 % CI)	OR (99 % CI)	OR (99 % CI)
		*n* = 2874	*n* = 3425	*n* = 3808
Recall e-cig supermarket	**2.56 (1.89 to 3.47)**	1.66 (0.95 to 2.89)	1.76 (0.94 to 3.29)	1.82 (0.99 to 3.38)
Recall e-cig small shop	**2.89 (2.36 to 3.54)**	**1.93 (1.51 to 2.48)**	**1.96 (1.64 to 2.34)**	**1.92 (1.61 to 2.29)**
Recall internet e-cig ads	**2.02 (1.73 to 2.35)**	**1.72 (1.17 to 2.52)**	**1.90 (1.26 to 2.84)**	**1.76 (1.29 to 2.40)**
Recall other e-cig ads	**1.57 (1.33 to 1.85)**	1.00 (0.83 to 1.20)	1.10 (0.93 to 1.31)	1.12 (0.91 to 1.36)
Never smoked tobacco		**0.08 (0.05 to 0.12)**	**0.08 (0.05 to 0.12)**	**0.08 (0.05 to 0.12)**
Gender- male		1.24 (0.96 to 1.60)	1.11 (0.76 to 1.61)	1.10 (0.71 to 1.71)
Ethnic group-non-white		1.81 (0.75 to 4.36)	1.48 (0.63 to 3.47)	1.44 (0.63 to 3.29)
Age		**1.15 (1.08 to 1.22)**	**1.17 (1.10 to 1.24)**	**1.16 (1.09 to 1.24)**
FAS low		1	1	1
FAS medium		1.01 (0.76 to 1.35)	1.00 (0.78 to 1.27)	1.04 (0.81 to 1.33)
FAS high		1.14 (0.63 to 2.04)	1.16 (0.65 to 2.06)	1.16 (0.66 to 2.03)

Bold *p* < 0.01

Hosmer-Lemeshow chi square goodness of fit Adjusted model 1 = 6.6, *p* = 0.58 Adjusted model 2 = 4.42, *p* = 0.82

The consistency of these results across alternative methods of handling missing data in the dependent variable were explored. The adjusted model 2 in Table 2 shows the effect of recoding the dependent variable so that young people who have not heard of e-cigarettes are assumed not to have used them. The adjusted model 3 shows the effect of using multiple imputation to fully impute the dataset. Neither of these alternative methods alter the results.

### Recall of e-cigarette POS displays and intention to use e-cigarettes

When the various sources of e-cigarette promotion were individually regressed on intention to try e-cigarettes, they were all found to be significant predictors of intention to try e-cigarettes (Table 3 unadjusted model). In other words, recall of e-cigarette POS in small shops and supermarkets, recall of e-cigarette adverts online, and recall of other types of e-cigarette adverts all significantly increased the odds of intending to use e-cigarettes in the next 6 months. In the adjusted model 1 where all sources of e-cigarettes, previous smoking experience, previous e-cigarette use and demographic variables were included in the model, recall of e-cigarette POS in small shops and supermarkets both remained significant independent predictors of intention to try e-cigarettes.

**Table 3:** Logistic regression on intention to try e-cigarettes in next 6 months - adjusted for clustering within school

	Unadjusted model	Adjusted model 1	Adjusted model 2 recoded e-cig variable	Adjusted model 3 fully imputed m = 20
	OR (99 % CI)	OR (99 % CI)	OR (99 % CI)	OR (99 % CI)
		*n* = 2819	*n* = 3370	*n* = 3808
Recall e-cig supermarket	**3.60 (3.03 to 4.27)**	**1.59 (1.17 to 2.17)**	**1.60 (1.18 to 2.18)**	**1.70 (1.22 to 2.36)**
Recall e-cig small shop	**3.93 (2.76 to 5.60)**	**1.87 (1.04 to 3.35)**	**1.88 (1.04 to 3.38)**	**1.80 (1.08 to 2.99)**
Recall internet e-cig ads	**2.38 (1.51 to 3.76)**	1.39 **(**0.78 to 2.49)	1.44 (0.83 to 2.50)	1.50 (0.77 to 2.95)
Recall other e-cig ads	**2.01 (1.87 to 2.17)**	1.28 **(**0.76 to 2.17)	1.31 (0.77 to 2.24)	1.24 (0.74 to 2.09)
Never smoked tobacco		**0.32 (0.27 to 0.39)**	**0.33 (0.27 to 0.39)**	**0.32 (0.24 to 0.43)**
Tried e-cigarette		**16.60 (13.09 to 21.04)**	**20.01 (15.34 to 26.11)**	**19.91 (13.76 to 28.80)**
Gender- male		1.59 (0.68 to 3.72)	1.56 (0.66 to 3.72)	1.56 (0.73 to 3.33)
Ethnic group-non-white		1.39 (0.90 to 2.14)	1.33 (0.85 to 2.08)	1.24 (0.69 to 2.22)
Age		**1.16 (1.00 to 1.33)**	**1.16 (1.01 to 1.33)**	**1.16 (1.07 to 1.27)**
FAS low		1	1	1
FAS medium		1.16 (0.82 to 1.63)	1.16 (0.82 to 1.64)	1.14 (0.79 to 1.63)
FAS high		**1.56 (1.05 to 2.31)**	**1.58 (1.07 to 2.33)**	**1.50 (1.05 to 2.13)**

Bold *p* < 0.01

Hosmer-Lemeshow chi square goodness of fit model 1 chi square = 8.63, *p* = 0.37, model 2 chi square- = 3.45, *p* = 0.90

Never having smoked tobacco was a protective factor, with those who had never smoked tobacco being less likely to intend to try e-cigarettes. As might be expected, young people who had tried e-cigarettes in the past were more likely to intend to use them again. Young people who were older and from more affluent families were more likely to intend to try e-cigarettes when all the other variables are included in the model.

Alternative methods of handling missing data were explored in adjusted models 2 and 3 in Table 3 and neither changed the results of the analysis.

### Comparison with intention to smoke cigarettes

The analysis was then conducted with intention to smoke cigarettes as the dependent variable and exposure to tobacco POS as the explanatory variable. This was done in order to compare the magnitudes of the odds ratios with the e-cigarette analyses above.

As shown in Table 4 the different sources of cigarette brand exposure were individually regressed on ‘intention to try cigarettes’. In the unadjusted model recall of cigarettes in small shops and in supermarkets and recall of cigarette adverts on the internet predicted intention to use cigarettes in the next year.

**Table 4:** Logistic regression on intention to smoke cigarettes- adjusted for clustering within school

	Unadjusted model	Adjusted model 1	Adjusted model 2 recoded e-cig variable	Adjusted model 3 imputed model m = 20
	OR (99 % CI)	OR (99 % CI)	OR (99 % CI)	OR (99 % CI)
		*n* = 2824	*n* = 3363	*n* = 3808
Recall tobacco supermarket	**1.79 (1.05 to 3.06)**	0.92 (0.64 to 1.32)	0.95 (0.60 to 1.51)	0.97 (0.56 to 1.70)
Recall tobacco small shop	**2.74 (1.81 to 4.16)**	**1.96 (1.45 to 2.65)**	**2.19 (1.48 to 3.23)**	**1.98 (1.17 to 3.35)**
Recall internet tobacco ads	**1.53 (1.41 to 1.67)**	**1.14 (1.02 to 1.28)**	1.14 (0.95 to 1.36)	1.19 (0.92 to 1.53)
Never smoked tobacco		**0.06 (0.03 to 0.09)**	**0.05 (0.03 to 0.09)**	**0.05 (0.03 to 0.08)**
Tried an e-cigarette		**3.22 (1.74 to 5.95)**	**3.12 (1.66 to 5.89)**	**3.08 (1.79 to 5.29)**
Gender- male		0.88 (0.62 to 1.24)	0.94 (0.72 to 1.22)	0.99 (0.75 to 1.33)
Ethnic group-non-white		0.76 (0.36 to 1.61)	0.76 (0.52 to 1.10)	0.85 (0.47 to 1.53)
Age		1.23 (1.00 to 1.52)	**1.23 (1.04 to 1.46)**	**1.24 (1.07 to 1.43)**
FAS low		1	1	1
FAS medium		1.46 (0.95 to 2.24)	1.39 (0.85 to 2.27)	**1.44 (1.01 to 2.04)**
FAS high		1.72 (0.97 to 3.05)	1.72 (0.92 to 3.24)	**1.81 (1.03 to 3.18)**

Bold *p* < 0.01

Hosmer-Lemeshow chi square goodness of fit model 1 chi square = 6.88 *p* = 0.55. Model 2 chi square = 11.56, *p* = 0.17

In the adjusted model 1, recall of seeing cigarettes in small shops and on the internet both predicted intention to use cigarettes in the next year. The odds ratios are in the same order of magnitude as those for the previous analyses with e-cigarettes. Never having previously smoked tobacco was a protective factor with those who had never smoked less likely to intend to do so in the future. Having tried e-cigarettes was also associated with intention to smoke tobacco in the next year.

Alternative methods of handling missing data were explored in adjusted models 2 and 3 in Table 4. In the case of intention to smoke recoding the e-cigarette variable and imputation both altered the results making the effect of internet cigarette adverts not statistically significant and high family affluence and age significant. This makes the outcome of the analysis more similar to that for intention to use e-cigarettes. The effect of recall of tobacco in small shops on intention to smoke remains significant in all the alternative treatments of the missing data.

## Discussion

This is the first study to look at e-cigarette POS recall and e-cigarette use and intention to use amongst adolescents. Our results show that there is an association between e-cigarette use by adolescents and awareness of POS displays of e-cigarettes. Adolescents who recalled seeing e-cigarettes in small shops were more likely to have tried an e-cigarette and adolescents who recalled seeing e-cigarettes in small shops and supermarkets were more likely to intend to try them in the next 6 months.

The effect of POS displays on e-cigarette experience is small relative to the effect of previous cigarette smoking. That is, much more of the variance in the probability of intending to try an e-cigarette is explained by whether or not the young person had ever smoked cigarettes than by whether they recalled e-cigarette POS displays. However, the magnitude of the effect of POS recall on intention to try e-cigarettes is comparable to that of tobacco POS displays on intention to smoke cigarettes. There are many studies showing that exposure to tobacco POS displays influences susceptibility to smoking and/or smoking initiation [8, 10, 27] and these findings have been supported by studies illustrating the reversibility of these effects [12]. This comparison suggests that although the effect size is small, interventions that reduce exposure to e-cigarette displays may have a measurable effect on susceptibility to e-cigarette use and initiation at the population level.

This study found that recall of e-cigarette adverts from other sources (print media, billboards and TV) was not a significant predictor of previous e-cigarette use or intention to use in the adjusted models. This indicates that once recall of point of sale and internet e-cigarette adverts is included in the models, recalling seeing adverts in billboards, newspapers or magazines or on TV does not account for any additional variance in uptake of e-cigarettes by adolescents. Recall of e-cigarettes in small shops and supermarkets contributes independently to the prediction of intention to use e-cigarettes. Recall of e-cigarette displays in small shops and on the internet is associated with previous e-cigarette use. Thus, e-cigarette advertising may be influencing adolescents from two sources – the physical spaces in which they live their lives and their online experience.

Small shops may be important as a source of e-cigarette point of sale exposure because they are frequently visited by young people. They also represent part of young people’s formative environment. Display of e-cigarettes in local small shops may indicate normality and social acceptability to young people [28]. The presence of e-cigarettes in small shops also indicates accessibility which has been shown to influence intention to smoke [29].

The other key source of e-cigarettes advert exposure is the internet. A recent report on children’s use of online technologies in Europe highlights ‘how embedded the internet has become in young people’s lives’ and that ‘most young people find the internet to be a necessity for life in modern society’ ([30], p.28). In the US, the Pew research group found ‘92 % of teens report going online daily including 24 % who say they go online “almost constantly”’ ([31], p.16). It is, therefore, unsurprising that internet sources influence teenagers.

### Limitations

The data, in this study, are cross-sectional so no firm conclusions can be drawn regarding causality. Therefore, we do not know whether young people plan to try e-cigarettes because they have noticed e-cigarette displays in shops or on the internet, or whether they notice e-cigarette displays because they are favourably disposed towards trying e-cigarettes. However, even if e-cigarette experimentation makes users more likely to notice displays, seeing displays in the local environment may well further reinforce that inclination [8]. Furthermore, longitudinal studies of traditional tobacco cigarette use have shown that exposure to point of sale displays leads to changes in smoking susceptibility [9]. Longitudinal data for e-cigarettes will be available from the DISPLAY study [23] over the next three years. In this future analysis, having three different measures of e-cigarette advertising recall in the same model means that covariance attributable to a predisposition to notice e-cigarette adverts is controlled in the adjusted models.

Another limitation is that the sample is not representative of the population of Scotland, as a fully randomised sampling process was not employed and there was 13 % non-response to the survey. However, demographic variables have been included in the adjusted models to control for these factors.

### Implications for e-cigarette POS display

This is the first study to suggest that there is an association between young people’s exposure to and recall of POS displays for e-cigarettes and their stated intentions to use e-cigarettes. Longitudinal data, which will be provided by this study in the future, will allow us to establish whether this relationship is causal. The possibility that e-cigarette POS displays may be linked to likelihood of uptake by young people is of concern, given that forthcoming EU legislation will state that e-cigarettes should be an adult-only product. There is some evidence, from animal models, that nicotine has long term effects on the adolescent brain including permanent changes to connectivity and neurotransmitter receptivity in neural reward-systems. These are the brain circuits associated with addictive behaviours [32, 33]. Therefore young people’s exposure to e-cigarettes at POS should be critically examined and potentially reduced. At the same time, however, e-cigarettes have potential positive health benefits for adult smokers [34] and some organisations have called for them to be displayed and promoted prominently at POS for this reason [35]. This raises an interesting policy dilemma, which can only be resolved by weighing up the relative benefits and costs of different options for the regulation of e-cigarette promotions, including banning POS. To a certain extent the resolution of the policy implications of our findings must await further evidence regarding the long term consequences of e-cigarette use and dual use in young people. As is the case for tobacco, the health consequences of e-cigarette use may only be fully apparent from long term longitudinal analysis [36]. The policy implications will also depend on how the e-cigarette market matures. If regular use of e-cigarettes among young people stabilises at its current levels (<5 %) and no long term adverse consequences emerge then it may be unnecessary to regulate e-cigarette point of sale. In the event that there is a continuing upward trend in regular e-cigarette use among young people and there is evidence of young people transitioning to tobacco smoking or other adverse health consequences from e-cigarettes then the information presented here on the relative importance of POS and other sources of e-cigarette advertisement will be important in influencing policy development.

## Conclusions

Our findings suggest that young people’s recall of e-cigarettes POS displays in small shops is associated with use of, and intention to use, e-cigarettes. Further research is required on how to balance the promotion of e-cigarettes as a cessation aid while preventing the initiation of e-cigarette use among young people who have never smoked.
